# Design and Evaluation of a Scalable and Reconfigurable Multi-Platform System for Acoustic Imaging

**DOI:** 10.3390/s16101671

**Published:** 2016-10-11

**Authors:** Alberto Izquierdo, Juan José Villacorta, Lara del Val Puente, Luis Suárez

**Affiliations:** 1 Signal Theory and Communications Department, University of Valladolid, Valladolid 47011, Spain; juavil@tel.uva.es; 2 Mechanical Engineering Area, Industrial Engineering School, University of Valladolid, Valladolid 47011, Spain; lvalpue@eii.uva.es; 3 Civil Engineering Department, Superior Technical College, University of Burgos, Burgos 09001, Spain; luis.a.suarez.vivar@gmail.com

**Keywords:** MEMS array, scalable system, multi-platform framework, acoustic imaging

## Abstract

This paper proposes a scalable and multi-platform framework for signal acquisition and processing, which allows for the generation of acoustic images using planar arrays of MEMS (Micro-Electro-Mechanical Systems) microphones with low development and deployment costs. Acoustic characterization of MEMS sensors was performed, and the beam pattern of a module, based on an 8 × 8 planar array and of several clusters of modules, was obtained. A flexible framework, formed by an FPGA, an embedded processor, a computer desktop, and a graphic processing unit, was defined. The processing times of the algorithms used to obtain the acoustic images, including signal processing and wideband beamforming via FFT, were evaluated in each subsystem of the framework. Based on this analysis, three frameworks are proposed, defined by the specific subsystems used and the algorithms shared. Finally, a set of acoustic images obtained from sound reflected from a person are presented as a case study in the field of biometric identification. These results reveal the feasibility of the proposed system.

## 1. Introduction

In recent years, techniques for obtaining acoustic images have been developed rapidly. At present, acoustic images are associated with a wide variety of applications [1], such as non-destructive testing of materials, medical imaging, underwater imaging, SONAR, geophysical exploration, etc. These techniques for obtaining acoustic images are based on the RADAR (RAdio Detection and Ranging) principles, which form an image of an object from the radio waves that have been reflected on it [2]. RADAR systems require high-cost hardware and their application with people and specific materials is difficult, due to their low reflectivity. These are the reasons why acoustic imaging techniques, also called SODAR (Sound Detection and Ranging) techniques, were developed. These SODAR techniques, mainly based on the use of arrays, represent a simple, low-cost alternative for obtaining “acoustic images” of an object.

An array is an arranged set of identical sensors, excited in a specific manner [3]. Microphone arrays are a particular case, used in applications such as speech processing, echo cancellation, localization, and sound sources separation [4]. By using beamforming techniques [5], the array beam pattern can be electronically steered to different spatial positions, allowing the discrimination of acoustic sources based on their position.

The authors of this paper have experience in the design [6] and development of acoustic imaging systems, based in acoustic arrays, used in many different fields, such as detection and tracking systems [7,8], Ambient Assisted Living [9], or biometric identification systems [10,11,12]. The arrays used were ULA (Uniform Linear Array), formed by acoustic sensors distributed uniformly along a line. These arrays are simple, but limited to one dimension (azimuth or elevation) to estimate the spatial position of the sound source. In order to obtain spatial information in two dimensions, it is necessary to work with planar arrays with sensors distributed on a surface.

Working with planar arrays leads to an increase in both system complexity and space required by the acoustic sensors and the associated hardware, since the extension from a 1D array of N element, to a 2D array of N × N elements increases the number of the required channels in an order of N^2^. These factors are accentuated when the number of sensors to work with is large. In the design of a system for acquiring and processing signals from an acoustic array, it should be noted that costs and complexity are directly related to the number of channels/sensors of the system. A typical system to obtain acoustic images has four basic elements: sensors, signal conditioners, acquisition devices and signal processor. For the first three elements, system cost increases linearly with the number of channels, as each sensor needs a signal conditioner and an acquisition device.

Digital MEMS (Micro-Electro-Mechanical System) microphones include a microphone, a signal conditioner, and an acquisition device incorporated in the chip itself. For this reason, an acquisition and processing system based on MEMS microphone arrays is reduced to two basic elements: MEMS microphone and a processing system. This processing system is usually based on an FPGA (Field Programmable Gate Array), as it is a digital system with multiple I/O nodes. The integration of the microphone preamplifier and the ADC in a single chip significantly reduces costs, if compared with solutions based on analog microphones. This is one of the main reasons why MEMS microphones are used in acoustic arrays with a large number of channels. However, cost reduction is not the only advantage of working with MEMS microphones. This technology also reduces the space occupied by the system, which makes it feasible to build arrays with hundreds or even thousands of sensors.

Arrays of MEMS microphones are specially designed for acoustic source localization [13,14,15]; however, they are also used in other applications such as DOA (direction of arrival) estimation for vehicles in motion [16], speech processing [17,18], turbulence measurements [19], identifying geometric dimensions and internal defects of concrete structures [20], or acoustic imaging [21,22,23]. The system presented in this paper has two main characteristics/novelties: (i) it is scalable using several subarrays or modules, increasing the number of sensors used, and reconfigurable in the position and orientation of the modules; and (ii) its multi-platform framework is reconfigurable. These characteristics made our system flexible and suitable for many different applications. Many of the systems found on the literature show fixed solutions to particular problems, where the hardware is not scalable or the software is not reconfigurable as in the system that is proposed in this paper. The system shown in [22] is reconfigurable, i.e., in the position of its modules, but its acquisition framework, based on an USB port, limits the maximum number of sensors that could be added to the system. For its part, the system shown by Turqueti [16] is scalable; it allows multiple arrays to be aggregated, as the system detailed in this paper. However, our system has two advantages in comparison with Turqueti’s system: (i) it can be a standalone system, due to the use of a myRIO platform (embedded hardware with a FPGA and an ARM processor) as an acquisition and data processing system; and (ii) it allows for emitting sounds, making it an active system.

This paper presents in detail the framework of a system that acquires and processes acoustic images. This system is based on a multi-platform framework, where each processing task can be interchanged between the different levels of the framework. Thus, the system can be adapted to different cost and mobility scenarios by means of its reconfigurable framework. The paper also shows the evaluation of processing times of the algorithms involved in obtaining an acoustic image, including signal processing and wideband beamforming via FFT, in each subsystem of the framework. The hardware of the system is based on modules of 8 × 8 square arrays of MEMS microphones, allowing the system to be scalable, by means of the cluster of several modules. This paper also shows the acoustic characterization of the MEMS microphones of the square array, as well as a comparative analysis of the theoretical and measured beam patterns of one of the array modules and of some clusters formed by several array modules.

Section 2 describes the hardware setup of the system and the implemented software algorithms, both defined on the basis of the requirements stated. Section 3 presents the planar array designed for the system: it introduces MEMS microphones technology and characterizes the frequency response of the microphones used, and it characterizes the array module acoustically, obtaining its beam pattern and also the beam pattern of some clusters of modules. A reconfigurable acquisition and processing framework is proposed in Section 4, and its performance is analyzed for several scenarios. Section 5 presents a case study using the system for biometric identification tasks. Finally, Section 6 contains the conclusions and future research lines.

## 2. System Description

In this section, the requirements for the implementation of the acquisition and processing system for a 2D array, based on MEMS sensors, are analyzed. Then, the hardware chosen for the implementation is defined, and the processing algorithms to obtain an acoustic image using beamforming techniques are explained.

### 2.1. Requirements

The size of an acquisition and processing system for an array depends on the number of sensors and the set of processing algorithms. Thus, it is necessary to use a very low-cost technology per channel, in order to build a viable high dimensional array. Using MEMS microphones allows for a cost reduction of two main elements of the system: sensors and acquisition systems. Digital MEMS microphones need only one digital input to be read, although the received digital data must be processed to obtain the waveform signal. Most acquisition systems are based on FPGAs, which have a large number of digital inputs, between 40 and 1400 depending on the model; so, one FPGA can acquire as much channels as the number of digital inputs it contains. Besides, FPGA processing capacity allows the system to carry out simple operations with the acquired signals without increasing costs. The processing capacity of the FPGA is not enough to obtain the final acoustic image so it is necessary that another processor with higher capacity be joined to the FPGA.

The system must be modular and scalable. Thus, for applications that require a large number of channels, it would only be necessary to add modules of lower dimension instead of designing a new system with higher capacity. In this way, reusing arrays and their processing systems reduces costs. Extra arrays, with their acquisition subsystem, can always be added in order to build higher dimensional systems. The array modules can increase the dimension of the array, but they can also form different kinds of module configurations in order to obtain specific beam patterns, improving the performance of the array, and thus of the complete system. The use of modular subsystems implies the use of a central unit that joins the data from all the modules and controls them.

Finally, the tools and programming languages to be used should also be defined. As this system has different processing platforms, one solution can be the use of a specific language for each platform, but the use of a common programming language on all platforms is desirable.

### 2.2. Hardware Setup

#### 2.2.1. MEMS Array

**Figure 1:**
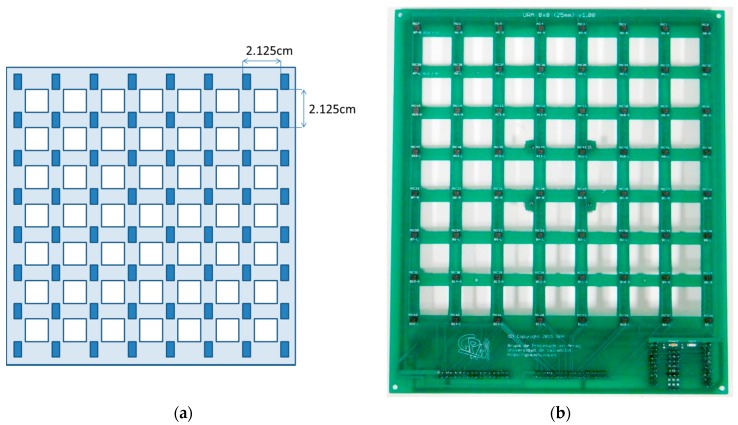
System microphone array: (**a**) sensor spacing; (**b**) system array.

The acoustic images acquisition system shown in this paper is based on a Uniform Planar Array (UPA) of MEMS microphones. This array, which has been entirely developed by the authors, is a square array of 64 (8 × 8) MEMS microphones that are spaced uniformly, every 2.125 cm, in a rectangular Printed Circuit Board (PCB), as shown in Figure 1. As can also be observed in Figure 1, the PCB where the MEMS microphones are placed has square gaps between the acoustic sensors, in order to make the array as light and portable as possible.

This array was designed to work in an acoustic frequency range between 4 and 16 kHz. The 2.125 cm spacing corresponds to λ/2 for the 8 kHz frequency. This spacing allows a good resolution for low frequencies, while avoiding grating lobes for high frequencies in the angular exploration zone of interest [10].

For the implementation of the array, MP34DT01 microphones of STMicroelectronics were chosen. They are digital MEMS microphones with a PDM (Pulse Density Modulation) interface and with a one-bit digital signal output, obtained using a high sampling rate (1 MHz to 3.25 MHz) [24,25,26]. The main features of these microphones are: low-power, omnidirectional response, 63 dB SNR, high sensitivity (−26 dB FS) and an almost flat frequency response (±6 dB in the range of 20 Hz to 20 kHz).

#### 2.2.2. Processing System

Taken into account the previous requirements, the hardware used to implement the system was selected. The design of a specific hardware was rejected due to the high cost of the design and the required time, so a search for a commercial solution was done.

MyRIO platform [27] has been selected as the base unit for this system. This platform belongs to the Reconfigurable Input-Output (RIO) family of devices from National Instruments that is oriented to sensors with nonstandard acquisition procedures, allowing low-level programming of the acquisition routines. Specifically, myRIO platform is an embedded hardware based on a Xilinx Zynq 7010 chip, which incorporates a FPGA and a dual-core ARM^®^ Cortex™-A9 processor. The FPGA has 40 lines of digital input/output, 32 of which are used as the connection interface with the 64 MEMS microphones of the array, multiplexing two microphones in each I/O line; while the other eight lines are used to clock generation and synchronization. The ARM processor is equipped with 256 MB of DDR3 RAM, 512 MB of built-in storage space, USB Host port, and Wi-Fi interface. All this hardware is enclosed in a small box (136 × 89 × 25 mm) that costs about $1,000.

**Figure 2:**
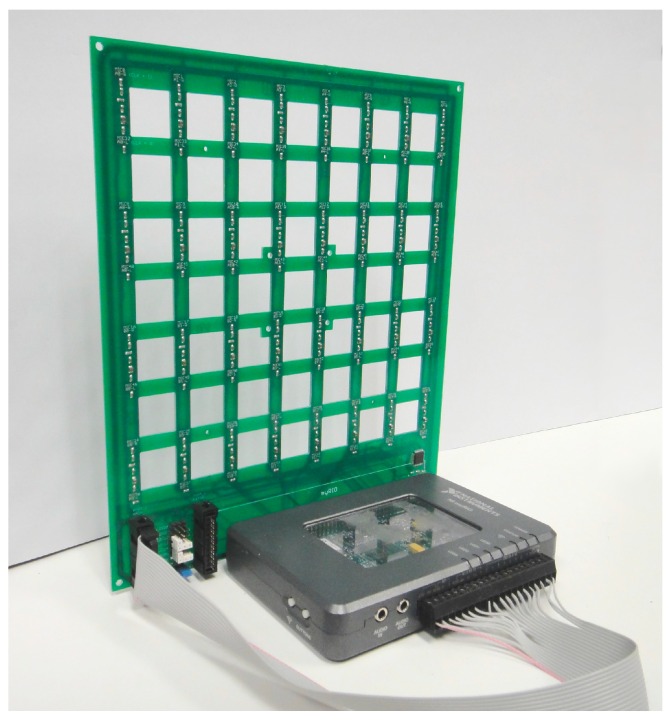
Array module with myRIO and MEMS array board.

The embedded processor included in myRIO is capable of running all the software algorithms to generate acoustic images, so it can be used as a standalone array module formed by a myRIO connected to a MEMS array board as shown in Figure 2. The acoustic images can be stored in the internal storage of myRIO or in an external disk connected through the USB port.

**Figure 3:**
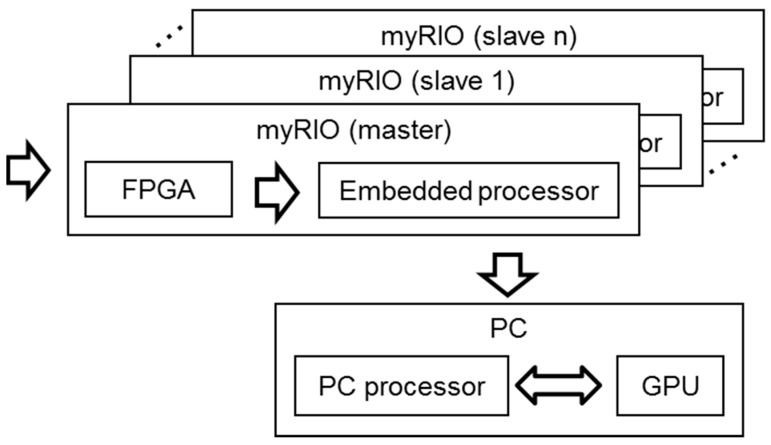
Global hardware setup.

Although myRIO can work as a standalone system, the lack of display means that it is usually controlled from a PC connected using a Wi-Fi interface. In a global hardware setup, as shown in Figure 3, the system includes a PC and one or more array modules. The PC performs three main functions:

- As user interface, the PC allows changing the system parameters and visualizing the acoustic images.
- As processing unit, the processors inside the PC could be used to execute the algorithms in order to obtain the acoustic images faster. Two processors are available in the PC: a general-purpose PC processor and a Graphical Processing Unit (GPU) included in the graphics card.
- As a control unit, a single PC can manage several myRIO platforms, each one associated to an array module. This feature allows clustering several modules for a proper operation of the system, which are synchronized between them using their I/O lines, where one myRIO is the master and the others are slaves.

### 2.3. Software Algorithms

**Figure 4:**
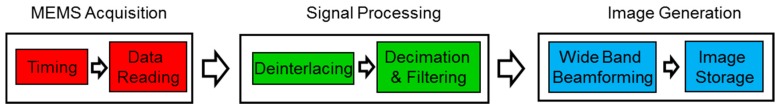
Software algorithms diagram.

The algorithms implemented in the system, shown in Figure 4, can be divided into three blocks: MEMS acquisition, signal processing, and image generation.

The programming language used is LabVIEW 2015, along with its Real Time, FPGA, and GPU modules, which allows developing applications on different hardware platforms like those used in the system: FPGA, Embedded Processor (EP), PC, and GPU. In addition, most of the developed algorithms can run on any of the platforms without reprogramming.

In the acquisition block, each MEMS microphone with a PDM interface, which internally incorporates a one-bit sigma-delta converter with a sampling frequency of 2 MHz, performs signal acquisition. So, each acquired signal is coded with only one bit per sample. This block is implemented in the FPGA, generating a common clock signal for all MEMS, and reading simultaneously 64 sensors signals via the digital inputs of the FPGA. These signals are stored in 64-bit binary words, where each bit stores the signal of each MEMS. Thus, the size of the data is minimal and the transfer rate is high.

In the signal processing block two routines are implemented: (i) Deinterlacing: Through this process, 64 one-bit signals are extracted from each binary word and (ii) Decimate & Filtering: Applying downsampling techniques, based on decimation and filtering [25], 64 independent signals are obtained and the sampling frequency is reduced from 2 MHz to 50 kHz.

**Figure 5:**
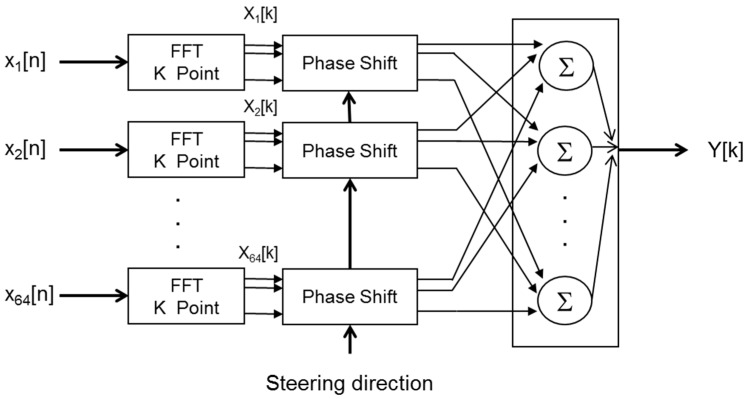
Wideband beamforming.

Finally, in the image generation block, based on wideband beamforming, a set of N × N steering directions are defined, and the beam former output are assessed for each of these steering directions. Wideband beamforming [3] computes the FFT of the MEMS signals x_i_[n]; multiplies, element by element, each FFT X_i_[k] by a phase vector, that depends on the steering direction and the sensor position; and finally takes the sum of the FFT shifted in phase, as shown in Figure 5. The images generated are then displayed and stored in the system.

## 3. MEMS Array Description and Characterization

The acronym MEMS refers to mechanical systems with a dimension smaller than 1 mm [28] manufactured with tools and technology arising from the integrated circuits (ICs) field. These systems are mainly used for the miniaturization of mechanical sensors. Their small size makes interconnection with other discrete components more difficult. Therefore, when ordered, they are supplied as part of an encapsulated micro-mechanical system composed by a sensor, a signal conditioning circuit and an electric interface [29].

### 3.1. MEMS Microphones Characterization

**Figure 6:**
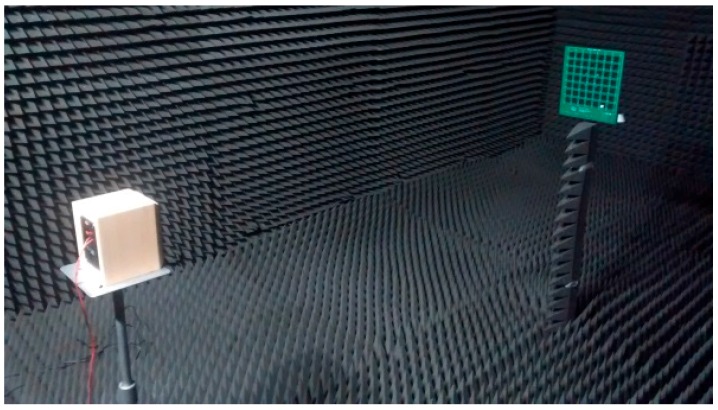
Arrangement of the components in the anechoic chamber.

An analysis of the frequency response of all MEMS microphones included in the array was performed. A sinusoidal 4 ms pulse, with a frequency changing between 2 and 18 kHz, was generated using a reference loudspeaker. Previously, the frequency response of the reference loudspeaker was calibrated using a measurement microphone (Behringer ECM 8000) and placing it in the same position as the array. Figure 6 shows the arrangement of the components used to perform this analysis. All measurements were performed in an anechoic chamber.

**Figure 7:**
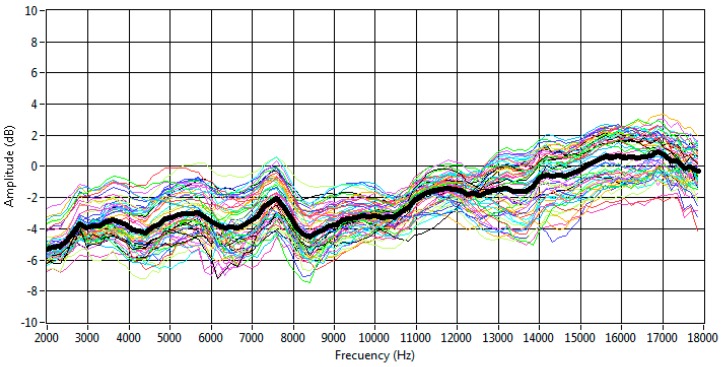
Frequency responses and averaged response of the MEMS microphones.

The frequency response of each MEMS sensor was obtained and normalized according to the loudspeaker’s response. Then, the average of the frequency responses was assessed. Figure 6 shows all the responses. It can be observed that the averaged frequency response is essentially flat, with a slight increase at high frequencies. This averaged response is bounded within a range of ±3 dB. Figure 7 also shows that the frequency response of MEMS sensors varies in a range of ±2 dB around the averaged value.

### 3.2. MEMS Array Characterization

#### 3.2.1. Acoustic Characterization of an Array Module

**Figure 8:**
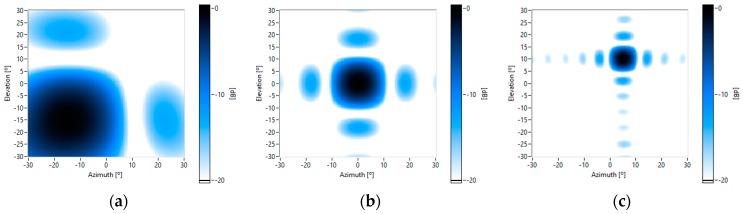
Theoretical beam patterns for different frequencies and pointing to different steering angles [azimuth, elevation]: (**a**) 4 kHz [−15°, −15°]; (**b**) 8 kHz and [0°, 0°]; and (**c**) 16 kHz and [5°, 10°].

Figure 8 shows some theoretical beam patterns of the UPA system, which are pointed towards several steering angles ([azimuth, elevation]): [−15°, −15°], [0°, 0°] and [5°, 10°], for several working frequencies: 4 kHz, 8 kHz and 16 kHz. It can be observed that: (i) as the frequency increases, the array angular resolution also increases, because the mainlobe beamwidth is reduced; and (ii) the sidelobe level is constant –at −13 dB for all frequencies. Figure 8b,c show that, for high frequencies, there are not grating lobes in the angular exploration zone of interest.

**Figure 9:**
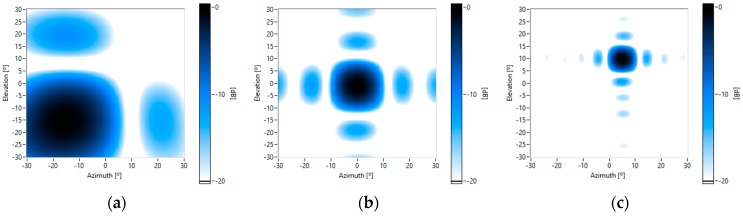
Measured beam patterns for different frequencies and pointing to different steering angles [azimuth, elevation]: (**a**) 4 kHz and [−15°, −15°]; (**b**) 8 kHz and [0°, 0°]; and (**c**) 16 kHz and [5°, 10°].

For the acoustic characterization of the MEMS array, a reference loudspeaker placed in different positions was employed to obtain its beam patterns. Beamforming was carried out with a wideband FFT algorithm, focused on the loudspeaker position. Figure 9 shows some of these beam patterns.

The measured beam patterns are very similar to the theoretical ones, which assume that the acoustic sensors are omnidirectional and paired in phases. Nevertheless, a more detailed analysis of the measured beam patterns shows: (i) there are more sidelobes with a level higher than −20 dB; and (ii) there is a very small displacement of the sidelobes, which are closer. These effects are because the gain of each microphone is slightly different for each frequency, as shown in Figure 7. This is the same effect as applying windowing techniques to the beamforming weight vector, which modifies the level and the position of the sidelobes. Thus, as the variations of the measured beam pattern, with respect to the theoretical one, are limited, it is not necessary to apply calibration techniques to the array.

#### 3.2.2. Acoustic Characterization of Array Clusters

The proposed system, characterized by its modularity and scalability, can group together multiple modules with 64 sensors to build clusters with a very large number of sensors, where their geometry and spatial properties could be adapted to specific application requirements.

As an example, the acoustic characterizations of three clusters geometries are shown:

- A row cluster, to increase the directivity in one direction.
- A square cluster, to increase directivity in two orthogonal directions.
- A star cluster, to implement special radiation beam patterns.

**Figure 10:**
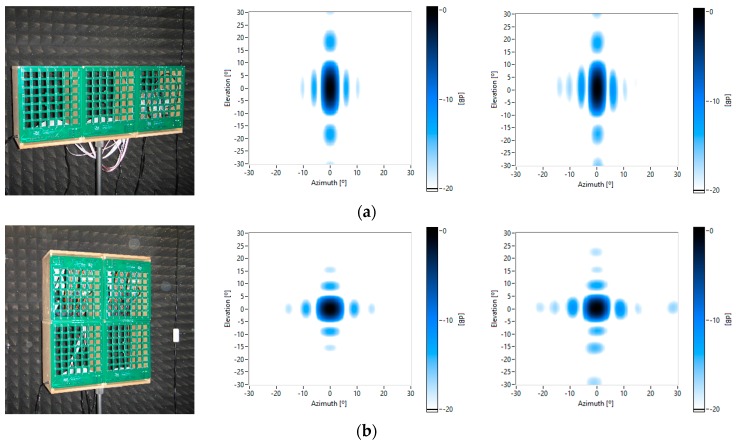

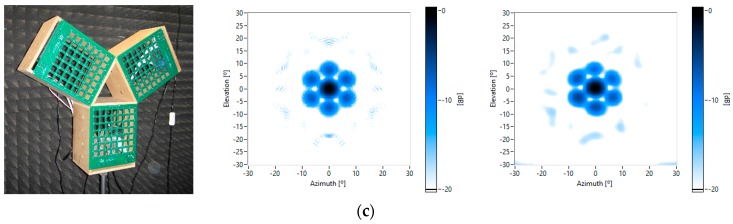
Cluster (col. 1), theoretical (col. 2) and measured beampatterns (col. 3) for 8 kHz, pointed to the broadside. Cluster geometries: row (**a**); square (**b**); and star (**c**).

Figure 10 shows the implemented cluster, the theoretical beam pattern, steered towards the broadside for 8 kHz, and the measured beam pattern.

The row cluster shows that the beamwidth in azimuth has been reduced by a factor of 3, increasing the angular resolution of the image in that direction. In the square cluster, the beamwidth in azimuth and elevation is halved. Finally, in the star cluster, a radial symmetrical pattern is achieved with a similar beamwidth in multiple directions.

## 4. Multiplatform Processing Framework

On the basis of the global hardware setup presented in Section 2, a multiplatform framework with four processing levels, each one implemented over a hardware platform, was defined. These processing levels are:

- Level 1 (L1) corresponds to the FPGA based on its capacity to carry out simple tasks of filtering and decimation. The parallelization degree is maximum and limited by the number of the FPGA resources (Look Up Tables, multipliers, DSP units, RAMs, etc.).
- Level 2 (L2) is based on an Embedded Processor (EP), such as an ARM processor that picks up the FPGA signals and carries out the first processing stages. It has limited memory as well as processing and storage capacity.
- Level 3 (L3) is based on a PC processor, such as an Intel Core i5/i7 with two to four cores. It is in charge of the main processing of the application, with medium cost and consumption. It has a high processing capacity, a great amount of memory (up to 64 Gb) and storage capacity based on a disk.
- Level 4 (L4) is formed by coprocessors, which can carry out massive FFT and lineal algebra operations, such as a Graphical Processing Unit (GPU), and they have from 200 to 1200 cores.

Processing time, parallelization degree and required memory must be analyzed for all the algorithms, needed to obtain an acoustic image, described in Section 2. The objective of this analysis is to determine the platforms/levels where these algorithms can be implemented, and the optimal distribution between the available algorithms and platforms.

The time required to transfer data between levels should also be taken into account. This transfer time, in many situations, can be similar to, or even greater than the algorithm processing time. Ideally, these transfers should be minimized, in order to process data on the same level and work with a one-way processing flow, i.e., the FPGA sends data to the EP, and it sends its data to the PC. When one level is used as a coprocessor, bidirectional flows are established, i.e., between the EP and the PC processor, through a TCP-IP interface, or between the PC processor and the GPU, using a PCIe interface.

### 4.1. Analysis Settings

**Figure 11:**
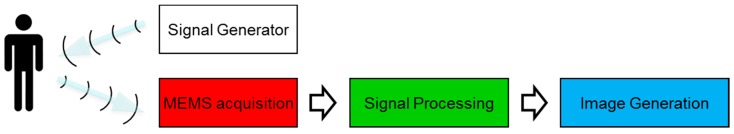
Processing block diagram.

In order to analyze the performance of the algorithms in each level, a work scheme, based on an active acoustic system, was defined. This system sends a multifrequency acoustic signal that reaches the person under test and then, the reflected signal is collected by the MEMS array. Finally, a multichannel signal is processed following the block diagram shown in Figure 11.

There are hardware constrains that make the implementation of some algorithms in every processing level unfeasible, i.e., MEMS acquisition can only be carried out in the FPGA, or image storage cannot be executed either in the FPGA or in the GPU. Table 1 shows the main algorithms used and the processing levels where they could be implemented.

The performance measurements have been carried out using the global hardware setup, with one array module, controlled by a PC. The selected PC is based on an i5 processor with four cores and 32 GB RAM, including a NVIDIA GTX 660 card with 960 cores. As algorithm parameters, an acquisition time of 30 ms, 256-point FFTs, and a grid of 40 × 40 steering directions have been assumed.

### 4.2. Performance Analysis

#### 4.2.1. Signal Processing

The time required to implement each of the algorithms included in the signal processing on the different levels are presented in Table 2.

Level 1 allows the implementation of all these algorithms using FPGA hardware resources, simultaneously with capture and signal processing without consuming additional processing time. Analyzing data from Levels 2 and 3, it can be observed that PC processing time is about 20 times lower than the time required by the EP. The times on Level 3 can be increased by transferring time from the EP to the PC for further processing. This transfer time was measured and its value is about 113 ms. Level 4, based on GPUs, was discarded because the algorithms required to perform deinterlacing and decimation/filtering are not available for this platform in LabVIEW.

#### 4.2.2. Image Generation

Table 3 shows the required processing times related with wideband beamforming and transfer times between the PC and the GPU, for the generation of an acoustic image.

Level 1 is discarded due to the fact that (i) the FPGA included in myRIO does not have enough memory to generate and store images; and (ii) the implementation of the beamforming algorithms requires FPGAs with a large number of slices which makes it more costly. Processing times on Level 2 are the longest and the memory capacity of the EP is limited, therefore, this level is also discarded.

In order to compare Levels 3 and 4, transfer times between the PC and the GPU should be considered. The results in Table 3 show that it is preferable to perform the beamforming on the PC. These results might seem contradictory, since the processing power of the GPU is higher than the PC’s. This is due to the following: (i) for 64 sensors, the GPU capacity of parallel processing is not totally used; and (ii) libraries included in LabVIEW-GPU are limited, which forces multiple data transfers between PC and GPU memories. The implementation of the beamforming algorithm in native code for the GPU and its subsequent invocation from LabVIEW would significantly reduce the overall processing time.

If the number of sensors of the system increases, by adding multiple modules of 64 sensors, the performed operations increase proportionately, taking advantage of the whole capacity of GPU parallel processing. In Table 4, the processing and transfer times versus the number of sensors is analyzed. If the number of sensors is larger than 128, GPU performance improves.

Image storage can be implemented in Levels 2 and 3 because embedded microprocessors and PCs have the capacity to store data. Embedded systems incorporate a low capacity internal disk and can use external high-capacity USB drives/disks. In the case of PCs, they include both high-capacity internal and external hard drives. As storage times are very dependent of the type and model of the used media, they were discarded for the analysis.

### 4.3. Framework Proposals

Depending on the level where the signal processing algorithm runs, there are three implementation options. In turn, for each of these possibilities, there are up to three options depending on where the wideband beamforming algorithm is implemented. Table 5 summarizes the different options to implement the acoustic imaging algorithms with their corresponding processing and transfer times for all feasible processing levels.

**Figure 12:**
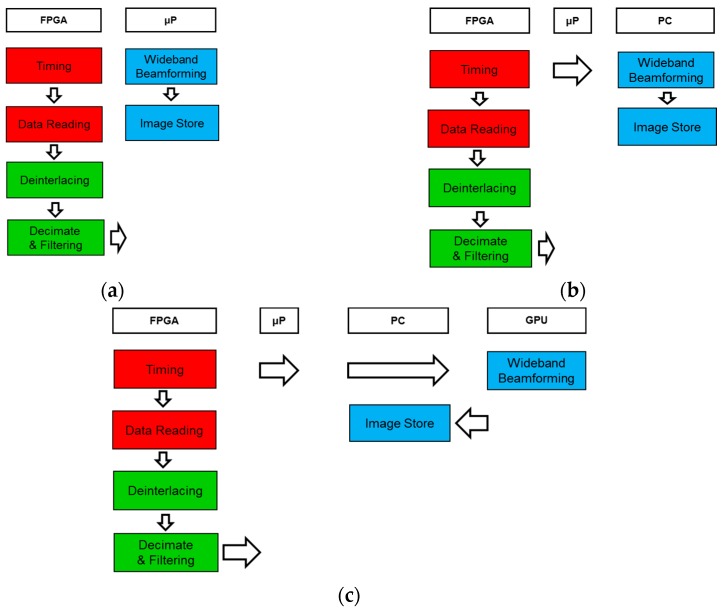
Embedded system (**a**); PC system (**b**); and PC system with GPU framework (**c**).

The frameworks that are based on the implementation of the signal processing algorithms through an embedded processor or a PC (grey columns) are discarded, as the FPGA allows the parallel implementation of the capture and signal processing. Thus, only three framework proposals (white columns) have been considered, associating each one to a particular use:

(1) Embedded system: The whole processing takes place in the embedded processor/FPGA without a PC, as shown in Figure 12a. This framework is optimal for applications that require portable systems and where the processing speed is not critical.
(2) PC system: The processing is shared between the FPGA and the PC. The embedded processor is used to control and transfer data between the PC and FPGA, as shown in Figure 12b. This framework presents the lowest processing time using 64 sensors. It is optimal for systems that require a short response time and/or a small number of sensors.
(3) PC system with GPU: The algorithms are implemented in the same way as in the previous framework excluding beamforming, which is implemented on the GPU, as shown in Figure 12c. This framework improves processing time as the number of sensors increases. It is the most versatile framework and it is optimal for systems that require a large number of sensors.

## 5. Case Study: Biometric Identification of People

The proposed system has multiple applications: localization and characterization of noise or vibration sources, spatial filtering and elimination of acoustic interference, etc. This case study is focused on biometric identification, as an extension of the previous system developed by the authors with a linear array of analog microphones [10]. This system could be used as an access control to enter in a medium-sized research department, where only several subjects have authorized access.

**Figure 13:**
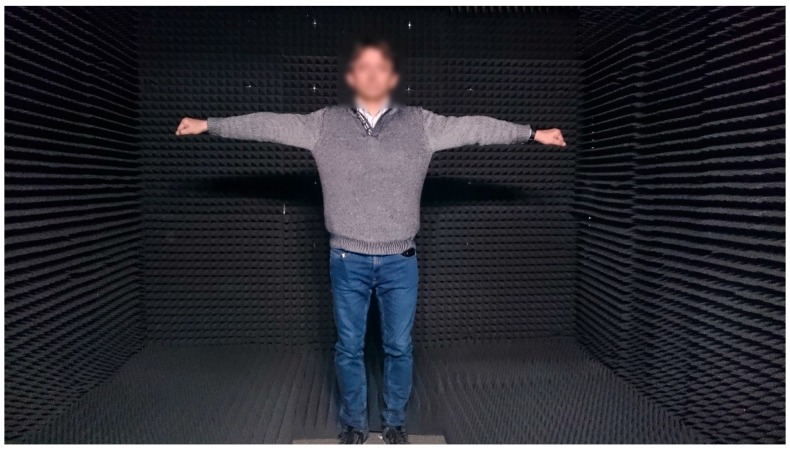
Example of a person under testing.

The biometric identification system is based on placing the person in front of the array and sending a multifrequency signal that is reflected on the person to be identified. The person and the system are placed inside an anechoic chamber in order to simplify the processing, but the chamber could be avoided if clutter removal techniques are used. The reflected signal is captured by the microphone array, obtaining several acoustic images for different ranges. The acoustic images are pre-processed to extract the information needed for further biometric identification. Figure 13 shows the image of a person under testing.

**Figure 14:**
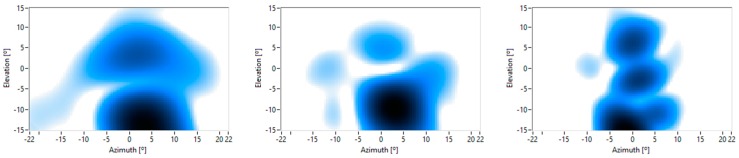
Acoustic images of a person with increasing frequencies, from left to right, at a constant range.

Figure 14 shows an example of some acoustic images obtained for a range of 2 m with a ±22° angle in the azimuth coordinate and ±15° angle in the elevation. It is observed that if the frequency increases, the spatial resolution improves and the main parts of the body could be discerned. The use of parameter extraction algorithms will improve the classification, as shown in the authors’ previous work, using 1D microphone arrays [12].

**Figure 15:**
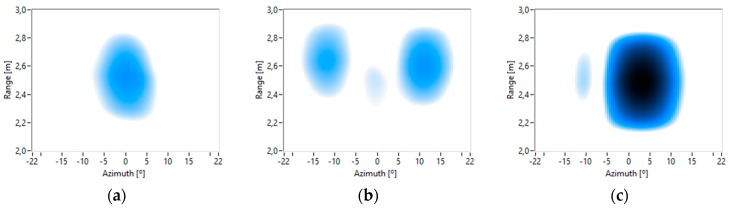
Acoustic images of a person at different elevations: (**a**) 5°; (**b**) 0°; (**c**) −10° with a constant frequency.

The range information can also be obtained from the captured images. Figure 15 shows images for a constant frequency and an elevation of 5°, 0° and −10°, with 0.5 m range intervals and a ±22° azimuth. Figure 15b shows that the torso is closer to the MEMS array than the arms. These results show that the designed system allows for the acquisition of 3D acoustic images.

## 6. Conclusions

A modular, scalable, multiplatform, and low-cost acquisition and processing system was designed to obtain acoustic 3D images. This system is based on a module with a myRIO platform and a planar array that consists of 64 MEMS microphones uniformly distributed on an 8 × 8 grid. The system can work with only one module or with a cluster of several of them, using the same PC unit. The system can be adapted to different cost and mobility scenarios, by means of its reconfigurable multi-platform framework, where each processing tasks can be interchanged between its different levels.

A digital MEMS microphone was selected as the acoustic sensor. This microphone allows the integration of a large number of sensors on a small sized board at low cost. An analysis of the frequency responses of these 64 MEMS microphones was carried out, obtaining: (i) a flat average response in the acoustic band, with a variation of ±3 dB; and (ii) dispersion between the responses lower than ±2 dB. The beampatterns of an array module and of different clusters of modules were also characterized, verifying that they indeed fit the theoretical models, so it is not necessary to calibrate the array.

Finally, a multiplatform framework and the necessary algorithms to obtain acoustic images from the data captured by each microphone were jointly defined. The processing time of the algorithms was evaluated on each platform. Based on these results, three different frameworks were defined for specific uses.

Thus, a versatile system for different applications, due to its modularity, scalability and reconfigurability, was designed to obtain acoustic images. Currently, the authors are using the system in the field of biometric identification, working on feature extraction and person identification from acoustic images.

## Figures and Tables

**Table 1:** Algorithms vs. processing level.

Algorithms	L1 FPGA	L2 EP	L3 PC	L4 GPU
MEMS acquisition	•			
Deinterlacing	•	•	•	
Decimation and filtering	•	•	•	
Wideband beamforming		•	•	•
Image storage		•	•	

**Table 2:** Processing and transfer times ^1^ for the signal processing tasks.

Algorithms	L1 FPGA	L2 EP	L3 PC	L4 GPU
Processing time: Deinterlacing	0	270.3	22.4	-
Processing time: Decimation/Filtering	0	354.1	9.7	-
Transfer time: EP→PC			113.0	
Total		624.4	145.1	

^1^ Time is expressed in ms.

**Table 3:** Processing and transfer times ^1^ for wideband beamforming.

Algorithms	L1 FPGA	L2 EP	L3 PC	L4 GPU
Processing time: Wideband beamforming	-	257.3	18.5	25.8
Transfer time: PC→GPU→PC				7.6
Total		257.3	18.5	33.4

^1^ Time is expressed in ms.

**Table 4:** Processing and transfer times ^1^ vs. number of sensors in the system.

# Sensors	PC	GPU		
	Processing Time: Wideband BF ^2^	Processing Time: Wideband BF	Transfer Time: PC⟷GPU	Total
64	18.5	25.8	7.6	33.4
128	49.1	40.7	8.1	48.8
256	100.2	72.9	8.7	81.6
512	198.6	138.8	11.2	150.0
1024	394.3	267.8	12.5	280.3

^1^ Time is expressed in ms; ^2^ BF: beamforming.

**Table 5:** Processing and transfer times ^1^ for acoustic imaging algorithms.

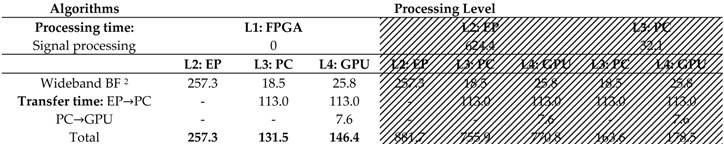

^1^ Time is expressed in ms; ^2^ BF: Beamforming.

## Abbreviations

The following abbreviations are used in this manuscript:

MEMS	Micro-Electro-Mechanical Systems
FPGA	Field Programmable Gate Array
FFT	Fast Fourier Transform
ULA	Uniform Linear Array
ADC	Analog-Digital Converter
DOA	Direction of Arrival
IC	Integrated Circuit
SNR	Signal to Noise Ratio
PDM	Pulse Density Modulation
I2S	Integrated Interchip Sound
PCM	Pulse Code Modulation
UPA	Uniform Planar Array
PCB	Printed Circuit Board
GPU	Graphical Processing Unit
RIO	Reconfigurable I/O
PC	Personal Computer
OS	Operative System
